# Supplementary material for: Integrated Assessment of Survival, Movement, and Reproduction in Migratory Birds: A Study on Evaluating Reinforcement Success
Source: Animals (Basel). 2024 Oct 30;14(21):3128. doi: 10.3390/ani14213128 (PMC11545394; doi:10.3390/ani14213128)
Supplement: Supplementary file 1 [file animals-14-03128-s001.zip › Supplementary Materials.pdf]

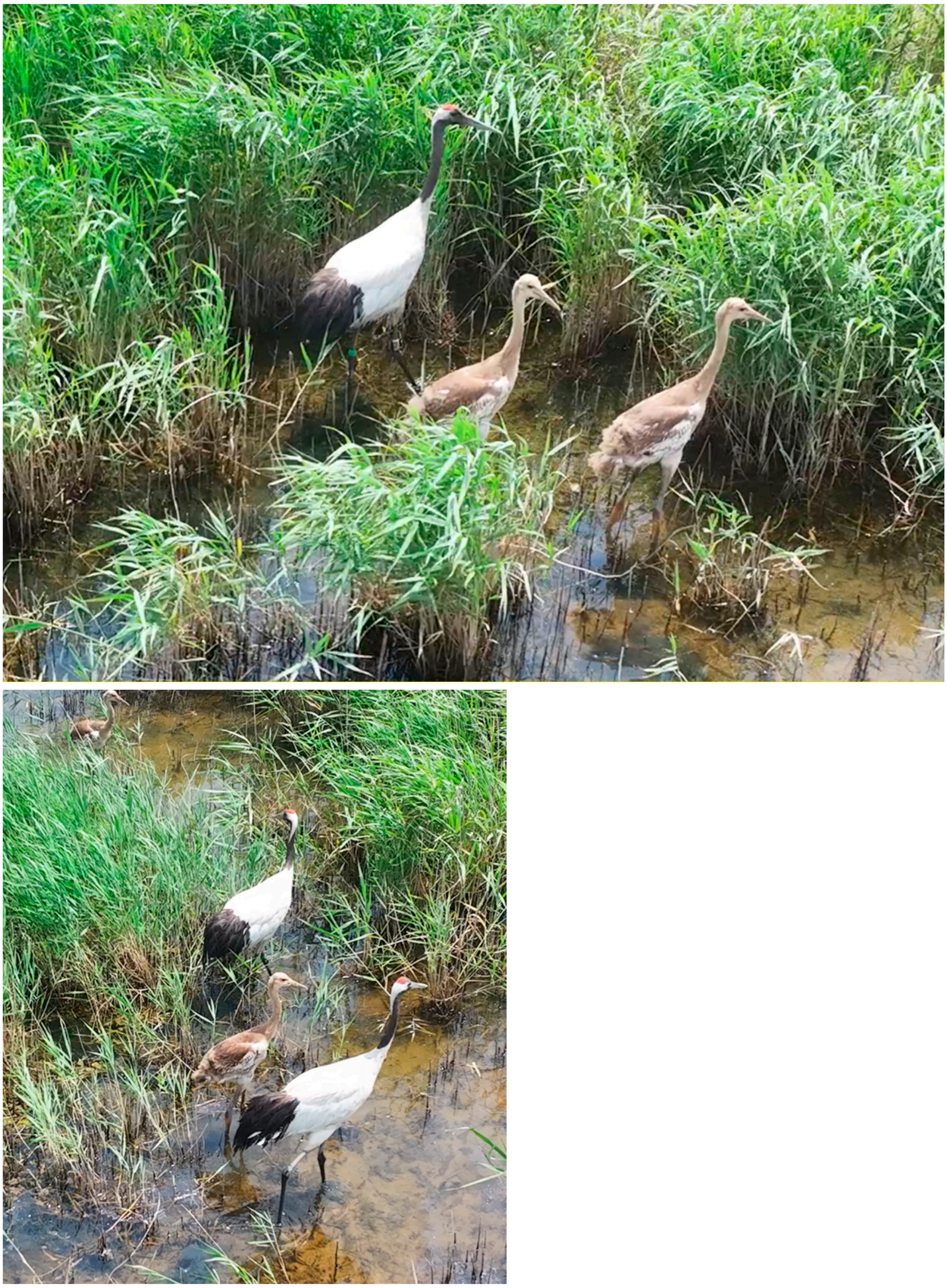

Figure S1 RS05, its mate, and their fledgling in the Shandong Yellow River Delta National Nature Reserve in 2024 (Photo by Guilin Hu).

Figure S2 R15: Left, with a blue ring on the leg, paired with a wild one (photo by Yongqiang Zhao)

Figure S3 W283: Left, paired with a wild individual in Heilongjiang Dazhan River Wetland National Nature Reserve before autumn migration, in 2012 (photo by Yanchang Gu); right, arrived in Japan on November, 2012 (photo by Nara).

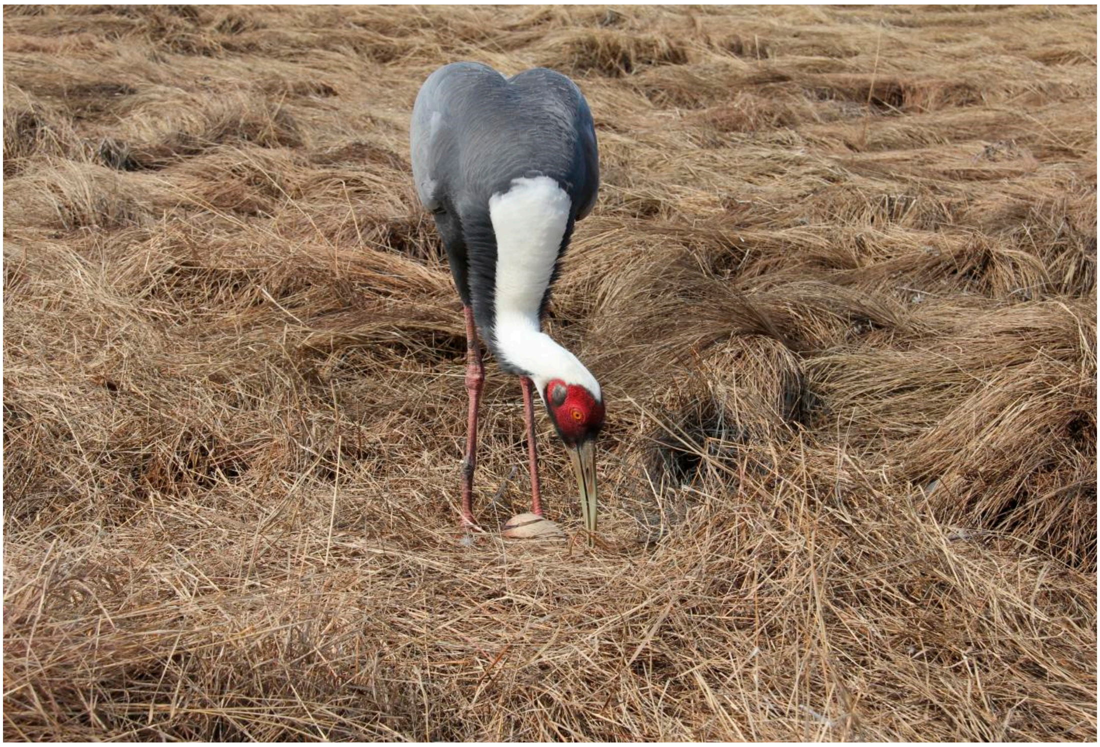

Figure S4 W283 with its eggs in the Heilongjiang Da Zhan River Wetland National Nature Reserve on April, 2013. (Photo by Yumin Guo)

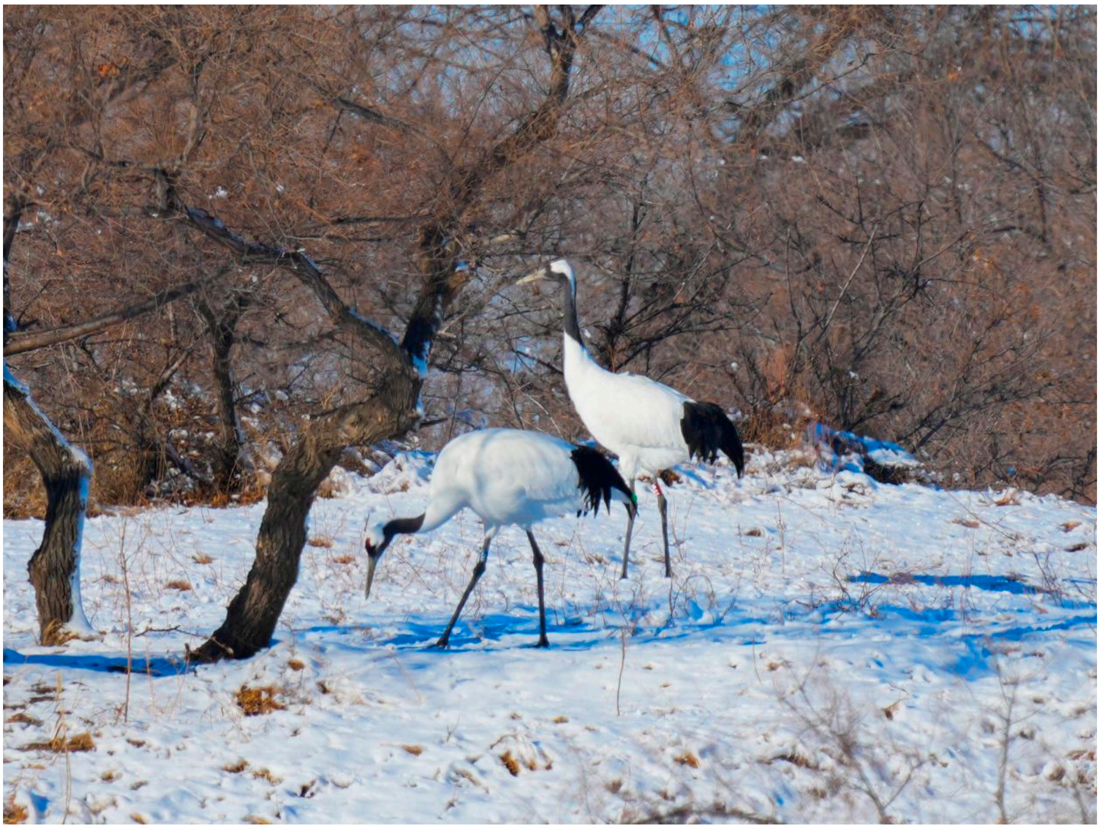

Figure S5 RA41 paired with a local released individual near its rescue site in Jilin Province on March, 2023.（Photo by Shengyu Pan）

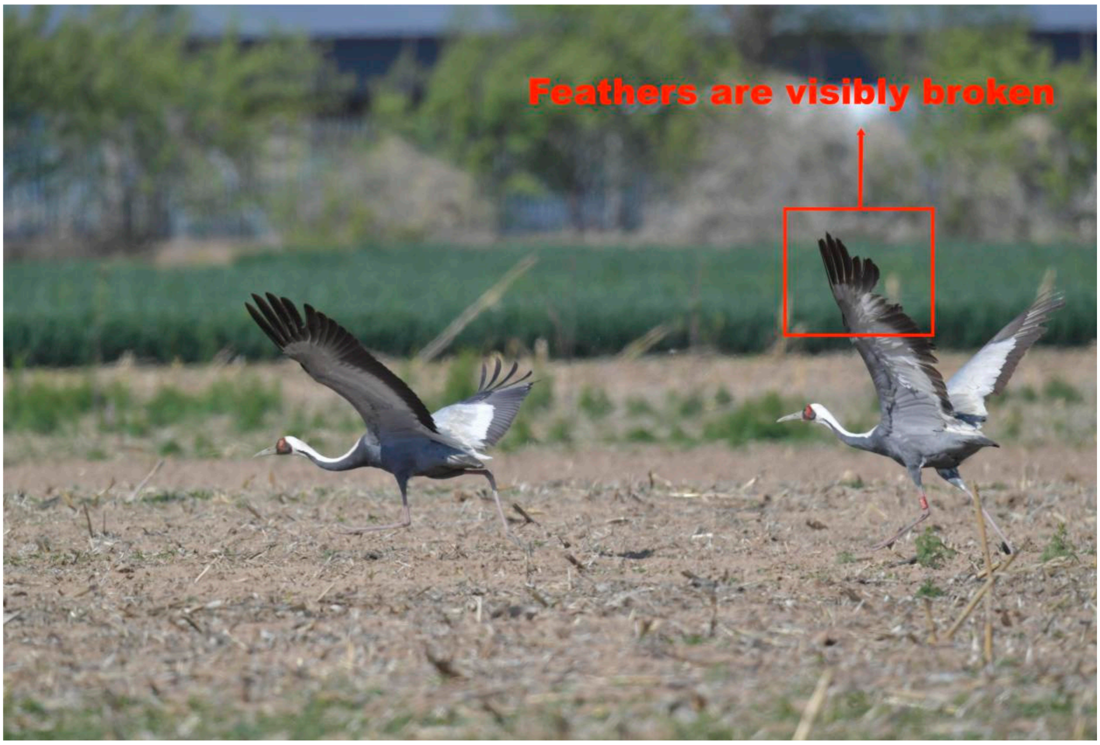

Figure S6 In Hebei Province, WS06 and its wild mate on 16 May, 2020.(photo by Yumin Guo)

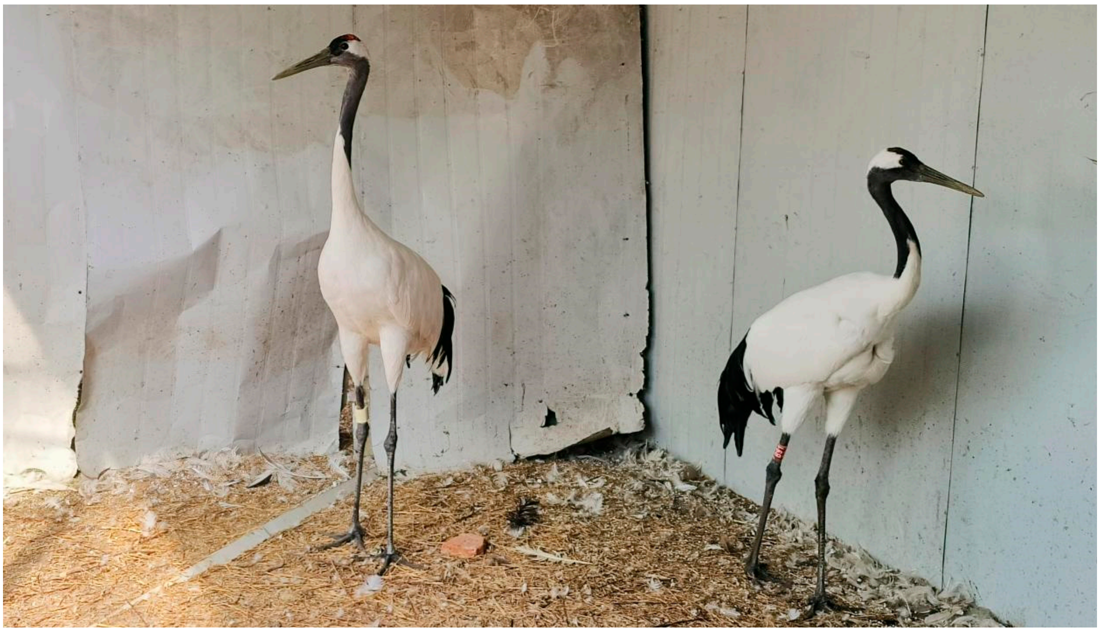

Figure S7 In Jilin Province, R200 and its wild rescued mate on May, 2024. (Photo by Yi Hao)
